# Hepatitis Secondary to an Over-the-Counter Appetite Stimulant: A Case Report

**DOI:** 10.7759/cureus.74289

**Published:** 2024-11-23

**Authors:** Shankar Neupane, Elizabeth Berg

**Affiliations:** 1 Pediatric Gastroenterology, New York City Health &amp; Hospitals/Lincoln Medical Center, Bronx, USA; 2 Pediatric Gastroenterology, Columbia University, New York, USA

**Keywords:** appetite stimulant, drug-induced liver injury, hepatitis, metabolic dysfunction-associated steatotic liver disease (masld), supplement, transaminase

## Abstract

We present the case of a previously healthy 11-year-old boy who developed hepatitis in the setting of regular intake of Apetamin. Apetamin is an appetite stimulant not approved by the United States Food and Drug Administration that contains cyproheptadine, lysine, and multiple B vitamins. It is illegally imported into the United States and can be purchased online. The patient was referred to the gastrointestinal clinic for evaluation of hepatitis based on screening laboratory testing collected during a routine healthcare visit. Laboratory workup revealed elevated transaminases six months after beginning Apetamin and positive anti-smooth muscle antibody. Liver enzymes improved following the discontinuation of Apetamin. The case highlights the potential of dietary supplements to cause hepatic injury and the importance of a thorough history of complementary alternative medication usage.

## Introduction

Prescription and non-prescription supplement use are relatively common for various reasons, including, but not limited to, induced weight gain or loss. In the United States, there are more than 100,000 dietary and herbal supplements available for purchase online or in stores, and several of them have been linked to drug-induced liver injury [1]. Supplements can affect the liver, causing hepatic injury, most commonly in the form of elevated liver enzymes [2]. Hepatitis can progress to liver dysfunction, fulminant hepatic failure, and, ultimately, liver transplantation or death [3]. Cyproheptadine, a first-generation antihistamine often an active constituent of appetite stimulants, can cause liver injury from time to onset of one to six weeks [4].

## Case presentation

An 11-year-old boy without a significant medical or surgical history was referred by his primary care provider to the gastrointestinal clinic for evaluation of elevated liver enzymes. He had a history of weight gain in the past with elevated blood glucose, which improved with lifestyle and dietary modifications. He was up to date on vaccines. Six months before the presentation, he complained of right upper quadrant pain for a few days without a history of jaundice, fever, or vomiting. There was no personal or family history of liver or metabolic disease. During his initial consultation, it was revealed that he had been using Apetamin, an appetite stimulant for the past two years. A physical examination revealed no pertinent positive findings. His body mass index was in the 51st percentile. The liver enzymes alanine aminotransferase (ALT) (two times the upper limit of normal) [5], aspartate aminotransferase, and alkaline phosphatase were found to be elevated 18 months before the presentation. Six months before the presentation, his aminotransferase levels were further elevated and ALT reached a peak level of 88 U/L during the initial evaluation in the gastrointestinal clinic; however, gamma-glutamyl transferase, albumin, bilirubin, and international normalized ratio were normal. He underwent serologic testing and abdominal ultrasonography to evaluate the differential diagnosis of hepatitis, including metabolic dysfunction-associated liver disease (MASLD), infectious, autoimmune, anatomic, and metabolic etiologies of hepatitis. The abdominal ultrasound was normal, and his laboratory workup was negative except for a borderline elevated anti-smooth muscle antibody of 1:20. He was advised to stop the use of Apetamin and avoid acetaminophen, alcohol, and sugar-sweetened beverages.

On a subsequent visit one month later, he had discontinued the use of Apetamin. His aminotransferase levels were decreased close to the normal range, suggesting that withdrawal of Apetamin eliminated a known hepatotoxic agent and led to improvement in hepatitis (Table 1).

**Table 1:** Liver enzyme trends.

Liver enzymes	Upper limit of the normal for boys (U/L)	09/2021	08/2022	02/2023	03/2023	06/2023
Alanine aminotransferase	25	51	70	88	29	36
Aspartate aminotransferase	35	56	59	52	29	34
Alkaline phosphatase	517	384	317	298	312	293

## Discussion

Hepatitis is frequently attributed to viral infections and MASLD. The patient was referred for evaluation of elevated liver enzymes, but it was Apetamin that caused reversible hepatitis. Apetamin has been reported to be associated with drug-induced autoimmune hepatitis [6]; however, in our case, the workup for autoimmune etiology was negative. Cyproheptadine, an active ingredient of Apetamin, is a US Food and Drug Administration-approved drug that has been reported to be associated with a cholestatic or mixed pattern of liver enzyme elevations [7]. Our patient had a hepatocellular pattern of liver enzyme elevation. While cyproheptadine is generally regarded as a safe drug, there have been rare cases where it was reported to cause drug-induced liver injury, occurring in 0.27-1.4 out of 1,000 patients [8]. The impact of cyproheptadine-induced liver injury is largely reported in case reports and case series. In a case series, 15 cases of liver injury possibly secondary to cyproheptadine use were reported, with three patients showing cholestatic hepatitis [9].

## Conclusions

This is a case of mild hepatitis with a normal evaluation and no classical explanation for the injury (i.e., infectious, anatomic, autoimmune). However, extensive history revealed the potentially toxic impact of an appetite stimulant purchased online. Several case reports or case series have indicated that the active ingredient cyproheptadine in Apetamin may be the reason for drug-induced liver injury. Patients need to be educated about the potential risk of liver injury from the use of appetite stimulants. The importance of history cannot be underestimated in identifying the cause of hepatitis, including the ingestion of potential toxins.
